# Longitudinal Outcomes of Dolutegravir-Based Antiretroviral Therapy in a Large Nigerian Programmatic HIV-1 Cohort, 2017–2019

**DOI:** 10.1093/ofid/ofag549

**Published:** 2026-08-28

**Authors:** Adam Abdullahi, Edun Martin, Asare Kwabena, Haruna Wisso Abdullahi, Ibrahim Musa Kida, Sophia Osawe, James Onyemata, Sani H Aliyu, Manhattan Charurat, Fatima-Murtala Ibrahim, Victor Adamu, Helen Omuh, Tim Efuntoye, Patrick Dakum, Alash’le Abimiku, Ravindra K Gupta

**Affiliations:** Takemi Program in International Health, Harvard Chan School of Public Health, Boston, Massachusetts, USA; Department of Medicine, Cambridge Institute of Therapeutic Immunology & Infectious Disease, Cambridge, UK; International Research Centre of Excellence, Institute of Human Virology Nigeria, Abuja, Nigeria; International Research Centre of Excellence, Institute of Human Virology Nigeria, Abuja, Nigeria; Department of Non-Communicable Disease Epidemiology, London School of Hygiene and Tropical Medicine, London, UK; International Research Centre of Excellence, Institute of Human Virology Nigeria, Abuja, Nigeria; Department of Infectious Diseases and Clinical Immunology, University of Maiduguri Teaching Hospital, Maiduguri, Nigeria; International Research Centre of Excellence, Institute of Human Virology Nigeria, Abuja, Nigeria; International Research Centre of Excellence, Institute of Human Virology Nigeria, Abuja, Nigeria; Addenbrooke's Hospital, Cambridge University Hospitals NHS Foundation Trust, Cambridge Biomedical Campus, Cambridge, UK; Institute of Human Virology, University of Maryland School of Medicine, Baltimore, Maryland, USA; International Research Centre of Excellence, Institute of Human Virology Nigeria, Abuja, Nigeria; Division of Global HIV & Tuberculosis, U.S. Centers for Disease Control and Prevention, Abuja, Nigeria; International Research Centre of Excellence, Institute of Human Virology Nigeria, Abuja, Nigeria; Division of Global HIV & Tuberculosis, U.S. Centers for Disease Control and Prevention, Abuja, Nigeria; International Research Centre of Excellence, Institute of Human Virology Nigeria, Abuja, Nigeria; International Research Centre of Excellence, Institute of Human Virology Nigeria, Abuja, Nigeria; Department of Medicine, Cambridge Institute of Therapeutic Immunology & Infectious Disease, Cambridge, UK; Africa Health Research Institute, Durban, South Africa; The Hong Kong Jockey Club Global Health Institute (HKJCGHI), Hong Kong Special Administrative Region, China

**Keywords:** antiretroviral therapy, Dolutegravir, HIV, Nigeria, treatment outcomes, virological suppression

## Abstract

In a retrospective Nigerian cohort of 33 982 people with HIV-1 initiating or switching to tenofovir, lamivudine, and dolutegravir, virological suppression reached 80%–82% among participants with available viral load data and 63%–65% by intention-to-treat analysis. Suppression was higher among switchers, while age and gender predicted suppression.

## Teaser

Among nearly 34,000 people receiving dolutegravir-based ART in routine HIV care in Nigeria, virological suppression reached approximately 80% at 12 months and 82% at 24 months among those with available viral load measurements. This large programmatic cohort provides important real-world evidence of sustained virological outcomes following dolutegravir implementation in Nigeria.

Increasing availability, accessibility, and uptake of antiretroviral therapy (ART) for HIV-1 treatment have led to improved life expectancy and quality of life for people with HIV (PHIV) [1, 2]. The remarkable global progress and gains of ART programs in reducing mortality and morbidity associated with HIV have been threatened by the considerable increase in drug resistance over the last decade [3–5]. This prompted the World Health Organization to update recommendations, and since 2019, the integrase strand transfer inhibitor dolutegravir (DTG) has been recommended as the preferred drug of choice for PLHIV, including children and adolescents [6].

Aligning with the UNAIDS 95-95-95 targets, which aim for 95% of PLHIV to know their status, 95% of people diagnosed with HIV to be established on ART, and 95% of those on ART attaining virological suppression [7], DTG-based ART has shown significant promise in improving ART outcomes and addressing challenges related to emerging drug resistance, especially in the face of the critical bidirectional effect of both HIV and sociocultural development in low middle income countries (LMIC) settings, such as Nigeria, where sociocultural factors are key players in the spread and management of HIV, with the epidemic in turn impacting the social and economic development. Observational outcomes of the systematic rollout and programmatic use of DTG in real-world settings are relatively limited but are starting to emerge with promising outcomes [8–11], especially across sub-Saharan Africa, where increased viral suppression rates and retention in care have been observed [12, 13]. In Nigeria, the HIV prevalence is 1.4% among adults aged 15–49 years [14] and 2.1% [15] at the national level, based on Bayesian predictive modeling, equating to approximately 2 million PLHIV.

Here, we assessed the programmatic virological suppression outcomes of the DTG rollout in an urban, large Nigerian population in Abuja, Nigeria, including factors associated with virological suppression using regression analysis.

## METHODS

The study population comprised a retrospective longitudinal cohort from Abuja, Nigeria. Data were obtained from the electronic medical record of the Nigerian HIV response program, supported by the US President's Emergency Plan for AIDS Relief (PEPFAR). Eligible participants initiated or transitioned to DTG in 2017–2019, had ≥6 months of follow-up after DTG initiation, and had at least one post-DTG viral load measurement. Participants were either ART-naïve or switched from a previous regimen. Variables extracted included age, sex, years on ART before DTG transition, and the most recent pre-transition viral load.

Viral load outcomes were assessed at 12 months (6–18 months) and 24 months (18–30 months) after DTG initiation, reflecting routine program monitoring schedules. These windows were selected to reflect routine viral load monitoring schedules within the Nigerian National HIV Program while accommodating the variability in testing commonly encountered in routine clinical practice. Where multiple viral loads were available within a follow-up window, the measurement closest to the target timepoint was selected. Viral loads obtained exactly 18 months after DTG initiation were assigned to the 24-month assessment window to avoid overlap (Supplementary Figure 1). Viral loads were categorized as virological suppression (when ≤50 copies/mL), low-level viremia (51–999 copies/mL), and virological non-suppression (≥1000 copies/mL) according to the predefined program viral load thresholds applied to the reported laboratory viral load results within the Nigerian National HIV Program.

Participant characteristics were summarized using frequencies, percentages, medians, and interquartile ranges (IQRs). Virological suppression rates were visualized using Sankey plots. We conducted univariable and multivariable modified Poisson regression analyses with robust sandwich standard errors and 95% confidence intervals (CIs) accounting for clustering by health facility to estimate risk ratios (RRs) for virological suppression at 12 and 24 months following initiation or transition to DTG. Analyses were restricted to participants with available viral load measurements (complete-case analysis) and adjusted for age and sex. Analyses were performed using R version 4.2.0. This project was reviewed under CDC human research protection procedures and determined to be nonresearch.

## RESULTS

### Patient Characteristics

Between 2017 and 2019, 33 982 participants aged 15 years or older were recorded as having started DTG-based ART, including 5171 (15.2%) ART-naïve participants and 28 811 (84.8%) who switched from a previous regimen. Most switchers (22 903 [79.5%] of 28 811) were previously receiving an efavirenz-based regimen with a median duration on ART of 4.4 years (IQR: 2.3–8.0) (Supplementary Table 1). The majority of participants (21 930 [65%] of 33 982) were women, and the median age at DTG initiation was 38 years (IQR: 32–45 years). Among the 28 811 participants who switched to DTG, recent viral load (within 12 months before DTG initiation) was available in 11 951 (41.5%), of whom 8722 (73%) had <50 copies/mL, 1638 (13.7%) had 50–999 copies/mL (low-level viremia), and 1591 (13.3%) had viral load ≥1000 copies/mL. A total of 10 360 (86.7%) had viral loads <1000 copies/mL, with a median time to measurement of 262 days (IQR: 217–310). Nearly all participants (99.6%) were on a tenofovir, lamivudine, and DTG (TLD)-based ART regimen.

### Virological Suppression Rates After 12 and 24 Months Following DTG Transition

**Figure 1.**
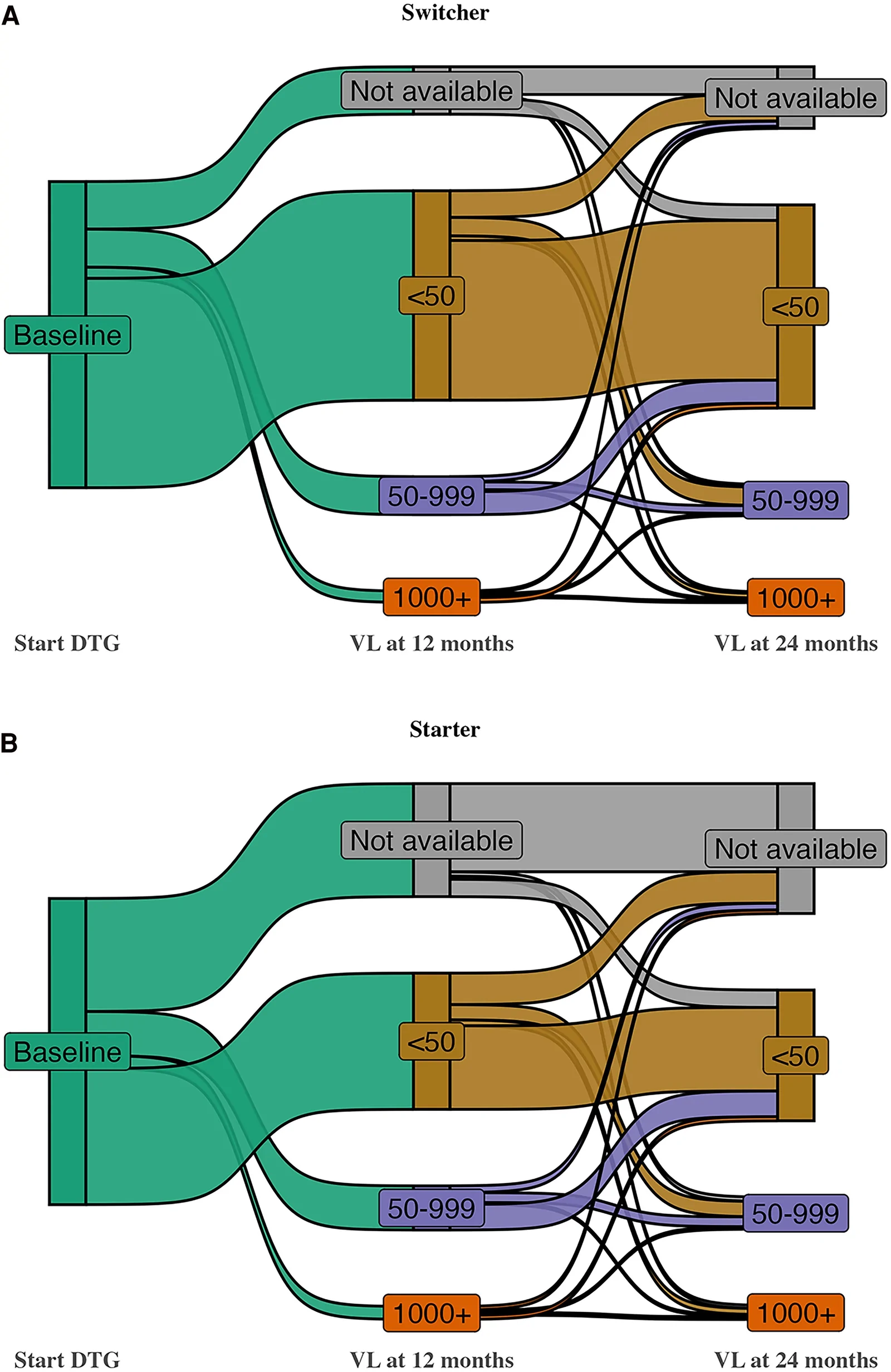
Longitudinal viral load trajectories among participants initiating dolutegravir-based therapy in Nigeria. Sankey plots illustrating longitudinal viral load dynamics among 33 982 participants who initiated dolutegravir-based therapy between 2017 and 2019. Panel *A* shows participants who transitioned from previous antiretroviral therapy regimens (“switchers”), while Panel *B* shows treatment initiators (“starters”). Viral load trajectories are shown at 12 months (n = 27 608) and 24 months (n = 25 990) following DTG initiation. Viral load categories were classified as virological suppression (<50 copies/mL), low-level viremia (50–999 copies/mL), and virological non-suppression (≥1000 copies/mL). Participants without available viral load measurements at each time point were classified as “not available.”

During follow-up, viral load data for assessment of virological outcomes at 12 months were available for 27 608 (81.2%) of 33 982 participants, including 24 345 (88.2%) switchers and 3263 (11.8%) initiators (Supplementary Table 1). Overall, per protocol, at 12 months, 22 006 (79.7%) of 27 608 were virally suppressed (HIV RNA <50 copies/mL). Suppression rates at 12 months were higher among switchers (19 706 [80.9%] of 24 345) compared with ART-naïve DTG initiators (2300 [70.5%] of 3263) (Figure 1). Low-level viremia (LLV) was observed in 4318 (15.6%) of 27 608, with lower LLV rates among switchers (3573 [14.7%] of 24 345) relative to starters (745 [22.8%] of 3263). Virological non-suppression (HIV 1 RNA ≥ 1000 copies/mL) occurred in 1284 (4.7%) of 27 608, also lower among switchers (1066 [4.4%] of 24 345) compared with initiators (218 [6.7%] of 3263) (Figure 1). These adjusted risk ratios should be interpreted as associations within this observational cohort and not as evidence that switching to DTG resulted in superior virological outcomes compared with ART initiation.

At 24 months, viral load data were available from 25 990 (76.4%) of the total 33 982 participants, with switchers comprising 23 012 (88.5%) and starters comprising 2978 (11.5%). Overall, 21 320 (82%) of 25 990 were virally suppressed, with higher rates among switchers (19 112 [83.1%]) compared with initiators (2208 [74.1%]). LLV was observed in 3458 (13.3%) participants, with a lower incidence among switchers (2922 [12.7%] of 23 012) compared with initiators (536 [18.0%]). Virological non-suppression occurred in 1212 (4.7%), with lower rates among switchers (978 [4.2%] of 23 012) compared with initiators (234 [7.9%]). Overall, higher suppression rates were observed at 24 months (82%) compared with 12 months (79.7%). It is noteworthy that in the intention-to-treat analysis, only 22 006 (64.7%) of 33 982 and 21 320 (62.7%) of 33 982 participants were virally suppressed at 12 months and 24 months, respectively. Viral load data availability declined over time, from 27 608 (81.2%) of 33 982 participants at 12 months to 25 990 (76.4%) of 33 982 at 24 months. A graphical overview of the longitudinal dynamics of viral load kinetics is shown in Figure 1. Baseline characteristics of participants with and without available viral load measurements at 12 and 24 months are presented in Supplementary Tables 2–5. Overall, demographic and program characteristics were broadly similar between participants with and without follow-up viral load data, although individuals without available viral load measurements were more frequently ART-naïve at DTG initiation and, when on ART, had a slightly shorter duration on ART before DTG transition.

### Factors Associated With Virological Suppression Following DTG Transition

**Figure 2.**
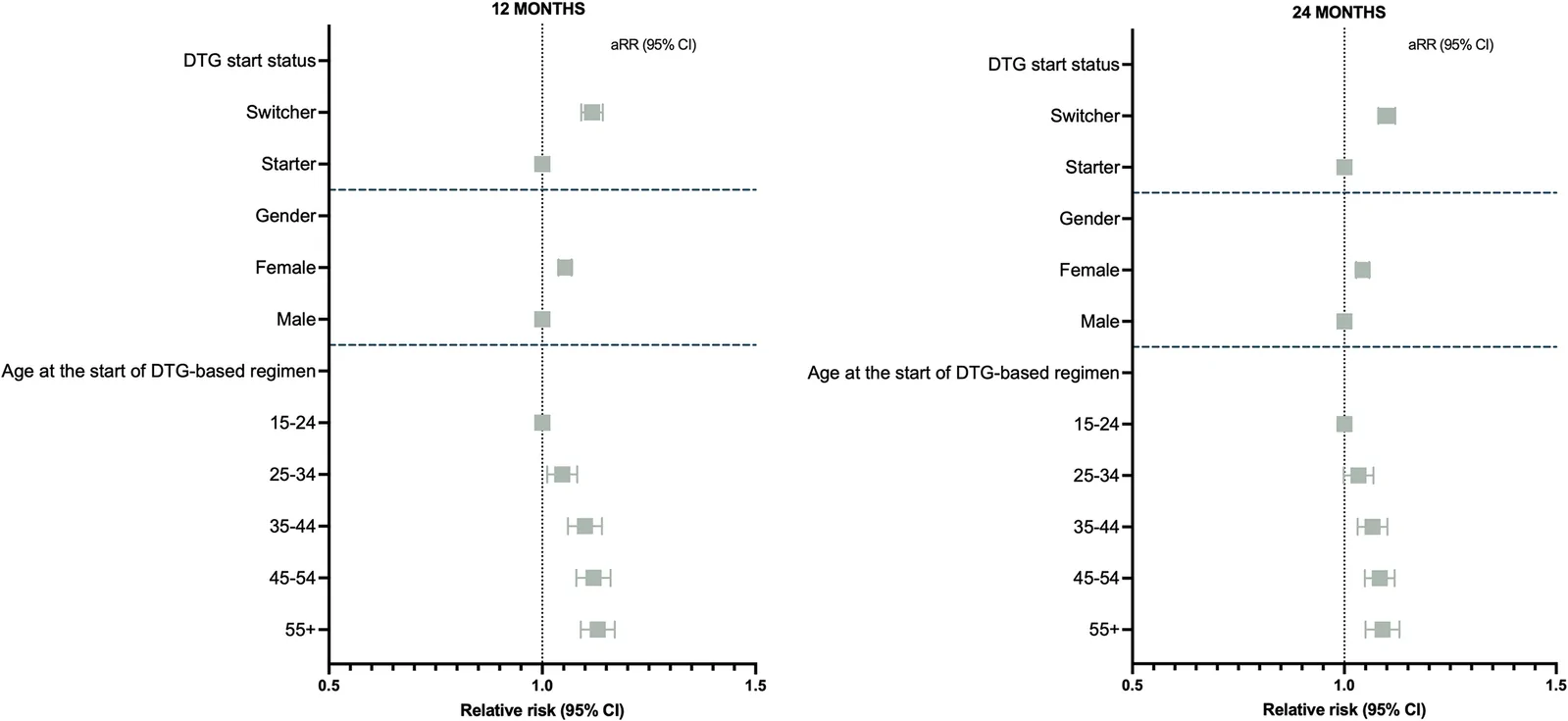
Factors associated with virological suppression following dolutegravir-based therapy. Forest plots showing predictors of virological suppression at 12 months (n = 27 608) and 24 months (n = 25 990) among participants with available viral load measurements following initiation of dolutegravir-based therapy. Virological suppression was defined as viral load <50 copies/mL. Adjusted risk ratios were estimated using modified Poisson regression models with robust sandwich standard errors. Variables included in the multivariable models included age, sex, and DTG start status. aRR, adjusted risk ratio; ART, antiretroviral therapy; CI, confidence interval; VL, viral load.

Of the 27 608 participants with available viral load measurements at 12 months, the median time to viral load testing was 376 days (IQR, 292–467) (Supplementary Table 1). In multivariable analyses, virological suppression was independently associated with older age, female sex, and transitioning to DTG from a previous ART regimen rather than ART initiation. The strongest association was observed among participants who transitioned from a previous regimen (aRR, 1.12; 95% CI, 1.09–1.14; *P* < .001; Figure 2 and Supplementary Table 6).

At 24 months, among the 25 990 participants with available viral load measurements, the median time to viral load testing was 687 days (IQR, 611–777). Older age, female sex, and transitioning from a previous ART regimen remained independently associated with virological suppression (Figure 2 and Supplementary Table 7), with treatment switchers continuing to demonstrate the highest likelihood of virological suppression (aRR, 1.10; 95% CI, 1.08–1.12; *P* < .001).

## DISCUSSION

Understanding the impact of the systematic rollout of DTG on virological outcomes is important for evaluating treatment outcomes under routine programmatic conditions and informing the continued optimization of HIV treatment programs in sub-Saharan Africa. Although DTG rollout began across several countries in the region in early 2017, longitudinal data describing virological outcomes following implementation remain limited, particularly in Nigeria. Here, we report follow-up outcome data from a large urban population who transitioned to DTG-based ART over 24 months of DTG-based ART administration by Nigeria's national HIV program. We found that over 12- and 24-month follow-up periods, suppression rates per protocol were 79.7% and 82%, respectively. We note the lack of viral load data at 12 and 24 months for around one in 5 participants, highlighting the various programmatic challenges. In this study, 15% of participants had low-level viremia at 12 months and 13% at 24 months, indicating opportunities to avert potential virological failure through adherence interventions. Encouragingly, only a small proportion of participants experienced virological failure across both time points (approximately 5%). However, when participants without available viral load measurements were classified as non-suppressed (intention-to-treat analysis), suppression estimates were 64.7% and 62.7% at 12 and 24 months, respectively.

We evaluated factors associated with virological suppression and found that participants who switched from a previous ART regimen were more likely to achieve virological suppression than ART-naïve initiators. However, these findings should be interpreted as adjusted associations within this observational cohort, as the 2 groups differed in baseline clinical characteristics, treatment history, and engagement in care. The lower suppression observed among initiators may reflect a higher burden of baseline NRTI or NNRTI resistance [3], as previously reported in South Africa and Nigeria [16, 17], although residual NRTI activity within TLD may also contribute [18]. We also observed that being female and older age are associated with a higher likelihood of virological suppression. This was not surprising, as older participants and females tend to have better health-seeking behaviors, especially in the African setting [18, 19].

Our study has several limitations. Although this large retrospective programmatic cohort provides important real-world evidence, approximately 20% of participants lacked viral load measurements at 12 or 24 months, resulting in incomplete outcome ascertainment. Missing viral load measurements likely reflect routine programmatic factors, including delayed laboratory testing, incomplete documentation, transfer of care, and loss to follow-up. Participants without available viral load measurements may have differed from those with documented outcomes, although the direction and magnitude of any resulting bias cannot be determined from the available data. Pre-transition viral load was defined using the most recent measurement within the preceding 12 months and may not fully reflect virological status at the time of DTG transition. To provide a more conservative assessment of treatment outcomes, we also reported intention-to-treat suppression estimates in which participants without viral load measurements were classified as non-suppressed, demonstrating lower suppression rates than the complete-case analyses and highlighting the importance of viral load coverage when interpreting programmatic outcomes.

We did not perform multiple imputation because viral load suppression was the primary study outcome, and the assumptions required for valid multiple imputation, particularly that outcome data were missing at random conditional on observed covariates, were unlikely to be met in this retrospective programmatic dataset. In addition, the routinely collected covariate data were insufficient to support robust multiple imputation or propensity score-based weighted analyses. Accordingly, our complete-case estimates should be interpreted as describing virological outcomes among participants with available viral load monitoring and not necessarily the entire eligible cohort. Finally, because this study relied on routinely collected programmatic data, assay-specific laboratory information was unavailable, and variability across laboratories may have resulted in minor misclassification of viral load measurements close to the assay detection threshold. Despite these limitations, this large programmatic cohort provides important real-world evidence on the outcomes following implementation of dolutegravir-based ART within routine HIV care in Nigeria.

In this large Nigerian programmatic cohort, virological suppression following implementation of dolutegravir-based ART was high among participants with available viral load monitoring at both 12 and 24 months, while high-level viremia (≥1000 copies/mL) was uncommon. Although incomplete outcome ascertainment and the absence of a non-DTG comparator limit causal interpretation and generalizability, these findings provide important real-world evidence supporting DTG implementation under routine HIV program conditions in Nigeria. Participants with persistent virological non-suppression should receive enhanced adherence support, ongoing viral load monitoring, and resistance testing where clinically indicated. Continued monitoring of viral load coverage, retention in care, and persistent viremia will be important to optimize long-term treatment outcomes as DTG-based regimens continue to expand across sub-Saharan Africa [16, 20, 21].

## Supplementary Material

ofag549_Supplementary_Data

## Supplementary Data

Supplementary materials are available at *Open Forum Infectious Diseases* online. Consisting of data provided by the authors to benefit the reader, the posted materials are not copyedited and are the sole responsibility of the authors, so questions or comments should be addressed to the corresponding author.
