# Supplementary material for: Longitudinal Outcomes of Dolutegravir-Based Antiretroviral Therapy in a Large Nigerian Programmatic HIV-1 Cohort, 2017–2019
Source: Open Forum Infect Dis. 2026 Aug 28;13(9):ofag549. doi: 10.1093/ofid/ofag549 (PMC13586762; doi:10.1093/ofid/ofag549)
Supplement: Supplementary file 1 [file ofag549_supplementary_data.pdf]

Supplementary table 1: Baseline characteristics of adults starting 1st line DTG-based ART

Characteristic	Overall N = 33,982[1]	Starter N = 5,171[1]	Switcher N = 28,811[1]
Age at the start of DTG-based regimen	38 (32-45)	35 (28-42)	39 (33-45)
Age at the start of DTG-based regimen			
15-24	1,904 (5.6%)	616 (11.9%)	1,288 (4.5%)
25-34	9,986 (29.4%)	1,905 (36.8%)	8,081 (28.0%)
35-44	13,243 (39.0%)	1,625 (31.4%)	11,618 (40.3%)
45-54	6,646 (19.6%)	767 (14.8%)	5,879 (20.4%)
55+	2,203 (6.5%)	258 (5.0%)	1,945 (6.8%)
Sex			
Male	11,916 (35.1%)	2,225 (43.0%)	9,691 (33.6%)
Female	22,066 (64.9%)	2,946 (57.0%)	19,120 (66.4%)
Level of ART health facility			
Tetiary	9,506 (28.0%)	782 (15.1%)	8,724 (30.3%)
Secondary	12,569 (37.0%)	1,675 (32.4%)	10,894 (37.8%)
Primary	3,685 (10.8%)	740 (14.3%)	2,945 (10.2%)
Faith Based	3,734 (11.0%)	463 (9.0%)	3,271 (11.4%)
One stop	1,889 (5.6%)	766 (14.8%)	1,123 (3.9%)
Unknown	2,599 (7.6%)	745 (14.4%)	1,854 (6.4%)
Years on ART before starting DTG	4.4 (2.3-8.0)	NA (NA-NA)	4.4 (2.3-8.0)
<1 year	2,490 (8.8%)	0 (NA%)	2,490 (8.8%)
1-5 years	12,970 (45.7%)	0 (NA%)	12,970 (45.7%)
5+ years	12,890 (45.5%)	0 (NA%)	12,890 (45.5%)
Available most recent viral load before starting DTG	12,189 (35.9%)	238 (4.6%)	11,951 (41.5%)
Most recent viral load (copies/mL) before starting DTG			
<50 copies per mL	8,846 (72.6%)	124 (52.1%)	8,722 (73.0%)
50-999 copies per mL	1,670 (13.7%)	32 (13.4%)	1,638 (13.7%)
1000+ copies per mL	1,673 (13.7%)	82 (34.5%)	1,591 (13.3%)
Days since most recent viral load before starting DTG	262 (217-310)	265 (220-317)	262 (217-309)
Most recent regimen before starting DTG			
TDF-3TC-EFV	22,903 (79.5%)	0 (NA%)	22,903 (79.5%)
AZT-3TC-NVP	5,403 (18.8%)	0 (NA%)	5,403 (18.8%)
AZT-3TC-EFV	136 (0.5%)	0 (NA%)	136 (0.5%)
TDF-3TC-NVP	109 (0.4%)	0 (NA%)	109 (0.4%)
Other PI or NNRTI based	260 (0.9%)	0 (NA%)	260 (0.9%)
Years on most recent regimen before starting DTG	2.70 (1.07-5.10)	NA (NA-NA)	2.70 (1.07-5.10)
<1 year	6,861 (23.8%)	0 (NA%)	6,861 (23.8%)
1-5 years	14,474 (50.2%)	0 (NA%)	14,474 (50.2%)
5+ years	7,476 (25.9%)	0 (NA%)	7,476 (25.9%)
DTG regimen			
TDF-3TC-DTG	33,827 (99.5%)	5,126 (99.1%)	28,701 (99.6%)
ABC-3TC-DTG	147 (0.4%)	38 (0.7%)	109 (0.4%)
AZT-3TC-DTG	8 (0.0%)	7 (0.1%)	1 (0.0%)
Number of viral load tests within 12 months after starting DTG			
0	6,374 (18.8%)	1,908 (36.9%)	4,466 (15.5%)
1	22,277 (65.6%)	2,293 (44.3%)	19,984 (69.4%)
2+	5,331 (15.7%)	970 (18.8%)	4,361 (15.1%)
12-month follow-up viral load available	27,608 (81.2%)	3,263 (63.1%)	24,345 (84.5%)
12-month follow-up viral load (copies/mL)			
<50 copies per mL	22,006 (79.7%)	2,300 (70.5%)	19,706 (80.9%)
50-999 copies per mL	4,318 (15.6%)	745 (22.8%)	3,573 (14.7%)
1000+ copies per mL	1,284 (4.7%)	218 (6.7%)	1,066 (4.4%)
Days to 12-month follow-up viral load	376 (292-467)	392 (287-485)	373 (292-462)
Concordance between 12 and 24 month viral load tests			
Both not available	4,095 (12.1%)	1,488 (28.8%)	2,607 (9.0%)
Only 12 month available	3,897 (11.5%)	705 (13.6%)	3,192 (11.1%)
Only 24 month available	2,279 (6.7%)	420 (8.1%)	1,859 (6.5%)
Both available	23,711 (69.8%)	2,558 (49.5%)	21,153 (73.4%)
Number of viral load tests within 24 months after starting DTG			
0	7,992 (23.5%)	2,193 (42.4%)	5,799 (20.1%)
1	19,358 (57.0%)	2,186 (42.3%)	17,172 (59.6%)
2+	6,632 (19.5%)	792 (15.3%)	5,840 (20.3%)
24-month follow-up viral load available	25,990 (76.5%)	2,978 (57.6%)	23,012 (79.9%)
24-month follow-up viral load (copies/mL)			
<50 copies per mL	21,320 (82.0%)	2,208 (74.1%)	19,112 (83.1%)
50-999 copies per mL	3,458 (13.3%)	536 (18.0%)	2,922 (12.7%)
1000+ copies per mL	1,212 (4.7%)	234 (7.9%)	978 (4.2%)
Days to 24-month follow-up viral load	687 (611-777)	693 (609-789)	686 (611-776)

[1]Median (Q1-Q3); n (%)

Supplementary Table 2: **Adults starting 1st line DTG-based ART between 20: 12m VL available**

Characteristic	Overall N = 27,608[1]	Starter N = 3,263[1]	Switcher N = 24,345[1]
Age at the start of DTG-based regimen	38 (32-45)	35 (29-43)	39 (33-45)
Age at the start of DTG-based regimen			
15-24	1,353 (4.9%)	353 (10.8%)	1,000 (4.1%)
25-34	7,771 (28.1%)	1,165 (35.7%)	6,606 (27.1%)
35-44	11,096 (40.2%)	1,062 (32.5%)	10,034 (41.2%)
45-54	5,609 (20.3%)	530 (16.2%)	5,079 (20.9%)
55+	1,779 (6.4%)	153 (4.7%)	1,626 (6.7%)
Sex			
Male	9,573 (34.7%)	1,440 (44.1%)	8,133 (33.4%)
Female	18,035 (65.3%)	1,823 (55.9%)	16,212 (66.6%)
Level of ART health facility			
Tetiary	8,303 (30.1%)	484 (14.8%)	7,819 (32.1%)
Secondary	10,114 (36.6%)	1,029 (31.5%)	9,085 (37.3%)
Primary	2,833 (10.3%)	435 (13.3%)	2,398 (9.9%)
Faith Based	3,142 (11.4%)	318 (9.7%)	2,824 (11.6%)
One stop	1,329 (4.8%)	535 (16.4%)	794 (3.3%)
Unknown	1,887 (6.8%)	462 (14.2%)	1,425 (5.9%)

[1]Median (Q1-Q3); n (%)

Supplementary Table 3: **Adults starting 1st line DTG-based ART : 12m VL missing**

Characteristic	Overall N = 6,374[1]	Starter N = 1,908[1]	Switcher N = 4,466[1]
Age at the start of DTG-based regimen	36 (30-44)	34 (27-41)	37 (31-45)
Age at the start of DTG-based regimen			
15-24	551 (8.6%)	263 (13.8%)	288 (6.4%)
25-34	2,215 (34.8%)	740 (38.8%)	1,475 (33.0%)
35-44	2,147 (33.7%)	563 (29.5%)	1,584 (35.5%)
45-54	1,037 (16.3%)	237 (12.4%)	800 (17.9%)
55+	424 (6.7%)	105 (5.5%)	319 (7.1%)
Sex			
Male	2,343 (36.8%)	785 (41.1%)	1,558 (34.9%)
Female	4,031 (63.2%)	1,123 (58.9%)	2,908 (65.1%)
Level of ART health facility			
Tetiary	1,203 (18.9%)	298 (15.6%)	905 (20.3%)
Secondary	2,455 (38.5%)	646 (33.9%)	1,809 (40.5%)
Primary	852 (13.4%)	305 (16.0%)	547 (12.2%)
Faith Based	592 (9.3%)	145 (7.6%)	447 (10.0%)
One stop	560 (8.8%)	231 (12.1%)	329 (7.4%)
Unknown	712 (11.2%)	283 (14.8%)	429 (9.6%)

[1]Median (Q1-Q3); n (%)

Supplementary Table 4: Adults starting 1st line DTG-based ART: 24m VL available

Characteristic	Overall N = 25,990[1]	Starter N = 2,978[1]	Switcher N = 23,012[1]
Age at the start of DTG-based regimen	39 (33-45)	36 (29-43)	39 (33-45)
Age at the start of DTG-based regimen			
15-24	1,225 (4.7%)	312 (10.5%)	913 (4.0%)
25-34	7,219 (27.8%)	1,029 (34.6%)	6,190 (26.9%)
35-44	10,516 (40.5%)	986 (33.1%)	9,530 (41.4%)
45-54	5,349 (20.6%)	503 (16.9%)	4,846 (21.1%)
55+	1,681 (6.5%)	148 (5.0%)	1,533 (6.7%)
Sex			
Male	9,155 (35.2%)	1,329 (44.6%)	7,826 (34.0%)
Female	16,835 (64.8%)	1,649 (55.4%)	15,186 (66.0%)
Level of ART health facility			
Tetiary	7,887 (30.3%)	480 (16.1%)	7,407 (32.2%)
Secondary	9,564 (36.8%)	974 (32.7%)	8,590 (37.3%)
Primary	2,506 (9.6%)	402 (13.5%)	2,104 (9.1%)
Faith Based	3,027 (11.6%)	290 (9.7%)	2,737 (11.9%)
One stop	1,172 (4.5%)	363 (12.2%)	809 (3.5%)
Unknown	1,834 (7.1%)	469 (15.7%)	1,365 (5.9%)

[1]Median (Q1-Q3); n (%)

Supplementary Table 5: Adults starting 1st line DTG-based ART: 24m VL missing

Characteristic	Overall N = 7,992[1]	Starter N = 2,193[1]	Switcher N = 5,799[1]
Age at the start of DTG-based regimen	36 (30-44)	33 (27-40)	37 (31-44)
Age at the start of DTG-based regimen			
15-24	679 (8.5%)	304 (13.9%)	375 (6.5%)
25-34	2,767 (34.6%)	876 (39.9%)	1,891 (32.6%)
35-44	2,727 (34.1%)	639 (29.1%)	2,088 (36.0%)
45-54	1,297 (16.2%)	264 (12.0%)	1,033 (17.8%)
55+	522 (6.5%)	110 (5.0%)	412 (7.1%)
Sex			
Male	2,761 (34.5%)	896 (40.9%)	1,865 (32.2%)
Female	5,231 (65.5%)	1,297 (59.1%)	3,934 (67.8%)
Level of ART health facility			
Tetiary	1,619 (20.3%)	302 (13.8%)	1,317 (22.7%)
Secondary	3,005 (37.6%)	701 (32.0%)	2,304 (39.7%)
Primary	1,179 (14.8%)	338 (15.4%)	841 (14.5%)
Faith Based	707 (8.8%)	173 (7.9%)	534 (9.2%)
One stop	717 (9.0%)	403 (18.4%)	314 (5.4%)
Unknown	765 (9.6%)	276 (12.6%)	489 (8.4%)

[1]Median (Q1-Q3); n (%)

Supplementary Table 6: Regression outcome at 12M post post-DTG use

	Table 1		Table 2				Table 3		
Characteristic	0 N = 5,598[1]	1 N = 22,010[1]	N	RR	95% CI	p-value	aRR	95% CI	p-value
Age at the start of DTG-based regimen			27,608						
15-24	376/1,353 (27.8)	977/1,353 (72.2)		—	—		—	—	
25-34	1,815/7,771 (23.4)	5,956/7,771 (76.6)		1.06	1.03, 1.10	<0.001	1.05	1.01, 1.08	0.011
35-44	2,111/11,096 (19.0)	8,985/11,096 (81.0)		1.12	1.08, 1.15	<0.001	1.1	1.06, 1.14	<0.001
45-54	998/5,609 (17.8)	4,611/5,609 (82.2)		1.12	1.08, 1.16	<0.001	1.12	1.08, 1.16	<0.001
55+	298/1,779 (16.8)	1,481/1,779 (83.2)		1.13	1.09, 1.18	<0.001	1.13	1.09, 1.17	<0.001
Sex			27,608						
Male	2,148/9,573 (22.4)	7,425/9,573 (77.6)		—	—		—	—	
Female	3,450/18,035 (19.1)	14,585/18,035 (80.9)		1.04	1.03, 1.06	<0.001	1.05	1.04, 1.07	<0.001
DTG start status			27,608						
Starter	962/3,263 (29.5)	2,301/3,263 (70.5)		—	—		—	—	
Switcher	4,636/24,345 (19.0)	19,709/24,345 (81.0)		1.13	1.11, 1.16	<0.001	1.12	1.09, 1.14	<0.001

[1]n/N (%)

Abbreviations: CI = Confidence Interval, IRR = Incidence Rate Ratio

Supplementary Table 7: Regression outcome at 24M post post-DTG use

	Table 1		Table 2				Table 3		
Characteristic	0 N = 4,662[1]	1 N = 21,328[1]	N	RR	95% CI	p-value	aRR	95% CI	p-value
Age at the start of DTG-based regimen			25,990						
15-24	289/1,225 (23.6)	936/1,225 (76.4)		—	—		—	—	
25-34	1,446/7,219 (20.0)	5,773/7,219 (80.0)		1.05	1.01, 1.08	0.005	1.03	1.00, 1.07	0.058
35-44	1,806/10,516 (17.2)	8,710/10,516 (82.8)		1.08	1.05, 1.12	<0.001	1.07	1.03, 1.10	<0.001
45-54	856/5,349 (16.0)	4,493/5,349 (84.0)		1.09	1.05, 1.13	<0.001	1.08	1.05, 1.12	<0.001
55+	265/1,681 (15.8)	1,416/1,681 (84.2)		1.09	1.05, 1.13	<0.001	1.09	1.05, 1.13	<0.001
Sex			25,990						
Male	1,811/9,155 (19.8)	7,344/9,155 (80.2)		—	—		—	—	
Female	2,851/16,835 (16.9)	13,984/16,835 (83.1)		1.04	1.02, 1.05	<0.001	1.04	1.03, 1.06	<0.001
DTG start status			25,990						
Starter	768/2,978 (25.8)	2,210/2,978 (74.2)		—	—		—	—	
Switcher	3,894/23,012 (16.9)	19,118/23,012 (83.1)		1.11	1.09, 1.14	<0.001	1.1	1.08, 1.12	<0.001

[1]n/N (%)

Abbreviations: CI = Confidence Interval, IRR = Incidence Rate Ratio

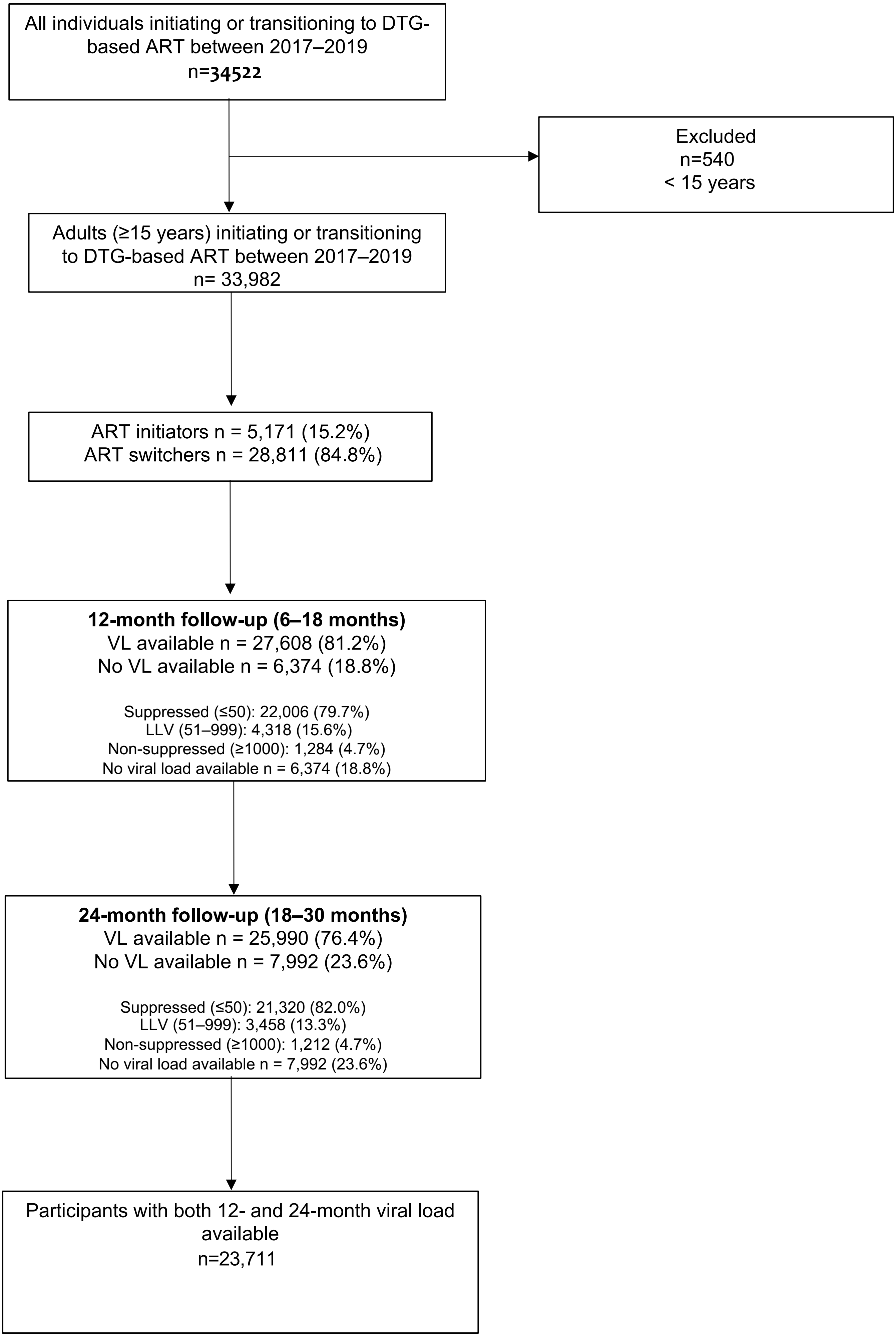

Supplementary Figure 1. Cohort derivation and availability of viral load outcome data. Flow diagram illustrating participant selection from the Nigerian National HIV Programme database, cohort derivation, and availability of viral load measurements within the predefined 12-month (6–18 months) and 24-month (18–30 months) follow-up windows following initiation or transition to dolutegravir (DTG)-based antiretroviral therapy between 2017 and 2019. LLV, low-level viraemia.
